# Supplementary material for: Induced endosymbiosis between a fungus and bacterium reveals a shift from antagonism to commensalism
Source: Nat Commun. 2025 Nov 28;16:10717. doi: 10.1038/s41467-025-65741-9 (PMC12663227; doi:10.1038/s41467-025-65741-9)
Supplement: Supplementary file 1 — Supplementary Information [file 41467_2025_65741_MOESM1_ESM.pdf]

Supplementary Figures for:

Induced endosymbiosis between a fungus and bacterium reveals a shift from antagonism to commensalism

Thomas Gassler[1*], Gabriel H. Giger[1], Anna Sintsova[1], Olivia X. Bossert[1], Alannah Holderbusch[1], Miriam Bortfeld-Miller[1], Benoit Dehapiot[2], Shinichi Sunagawa[1], Julia A. Vorholt[1*]

[1]Institute of Microbiology, ETH Zurich, 8093 Zurich, Switzerland

[2]ScopeM, ETH Zurich, 8093 Zurich, Switzerland

Present address: Gassler T., Department of Bioengineering and Bezos Centre for Sustainable Protein, Imperial College London, London, SW7 2AZ, United Kingdom

[*]Correspondence: t.gassler@imperial.ac.uk; jvorholt@ethz.ch

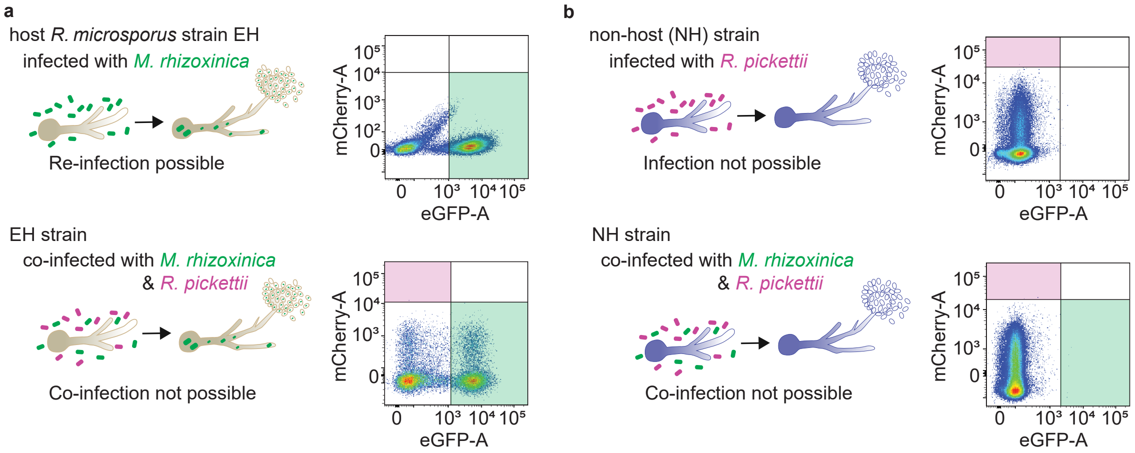

Supplementary Figure 1: Co-infection assay of the non-host (NH) strain and host (EH) strain with *R. pickettii* and *M. rhizoxinica*.

Re-infection of cured host strains (a) and NH strains (b) of *R. microsporus* with *M. rhizoxinica* and *R. pickettii*. **a** upper panel: re-infection of the EH-strain with *M. rhizoxinica* is possible (Gfp positive population of total arising spores were 42 %, 57 % and 73 %); **b** lower panel: co-incubation of *R. pickettii* together with *M. rhizoxinica* does not lead to co-infection (Gfp positive population of total arising spores were 41 and 61 %, no mCherry positive spores were obtained); **b**, co-incubation of the bacterial strains and the NH-strain shows that *R. pickettii* cannot infect the NH-strain under the tested conditions; upper panel: exemplary FACS analysis of NH-spores after co-cultivation with *R. pickettii* alone; lower panel: exemplary FACS analysis of NH-spores after co-cultivation with *R. pickettii* and *M. rhizoxinica*. Spores with a high mCherry signal were checked microscopically after FACS sorting and did not contain bacteria. Source data are provided as a Source Data file.

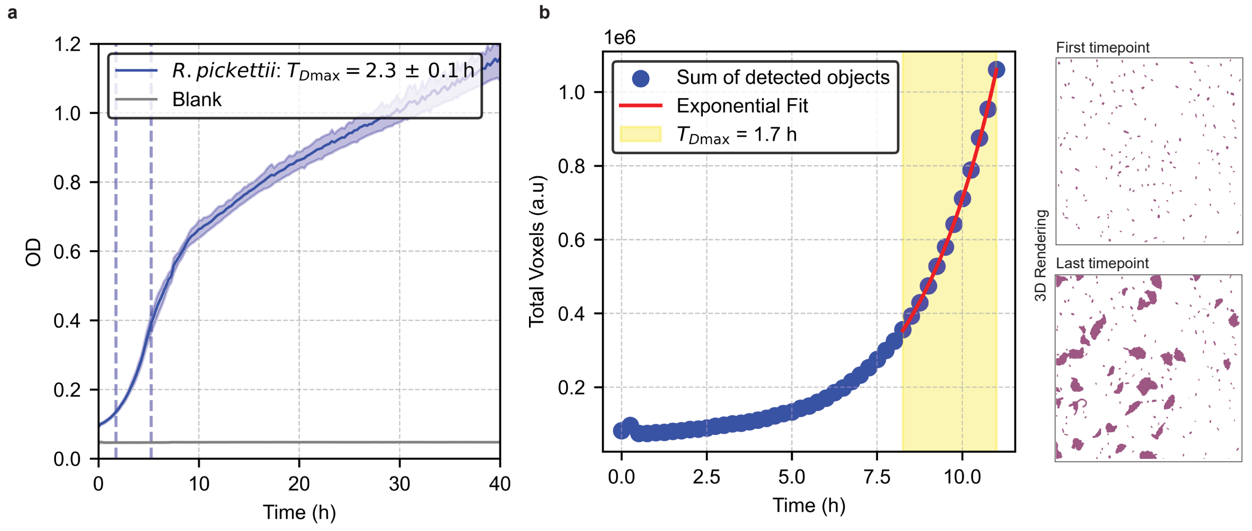

Supplementary Figure 2: Assessment of *R. pickettii* in vitro growth.

a, Determination of maximal doubling time T_{Dmax} in liquid medium. Cells were cultivated in 96-well plates with MGYM9 medium (glycerol as carbon source), (n = 12, average is depicted ± standard deviation; area between dashed lines marks time for μmax determination); **b**, determination of bacterial maximal doubling time T_{Dmax} by cultivation on agar, shown is the average of the obtained growth rate from all detected objects (right side in panel b) within the field of view with red line indicating the exponential fit (yellow area marks time for μ_{max} determination). Source data are provided as a Source Data file.

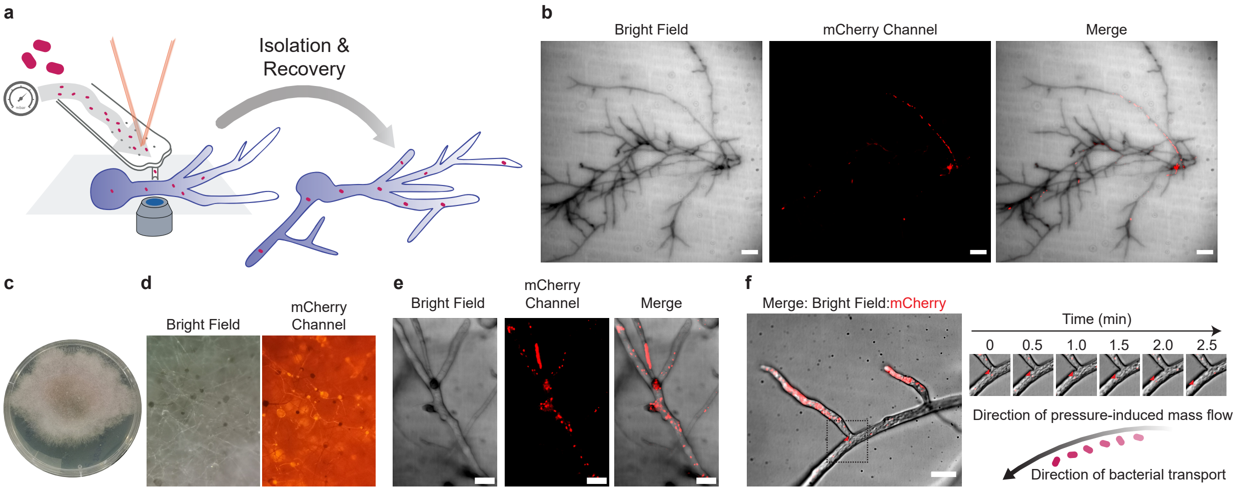

Supplementary Figure 3: Colonization of bacteria inside vegetative hyphae and movement.

a, Scheme of injection followed by isolation and recovery; **b**, Recovered germling 16 hours after injection; **c**, Recovered germling on plate showing red coloring of vegetative mycelium by introduced *R. pickettii*; **d**, Image of vegetative mycelium on plate taken by stereomicroscope shows regions of bacteria rich zones on macroscopic scale; **e**, Mycelium from regeneration plate shows heterogeneity in bacterial colonization; **f**, Movement of bacteria inside recovered occurs passively along direction of pressure-induced cytosolic mass flow, image shows bacteria rich zones at apical sites of growing hyphae and movement of single bacterial cells in cropped images over time; Bars: b = 100 µm, e = 20 µm, f = 30 µm, experiments were individually repeated with similar results three times. Source data are provided as a Source Data file and imaging data on zenodo (ref[69]).

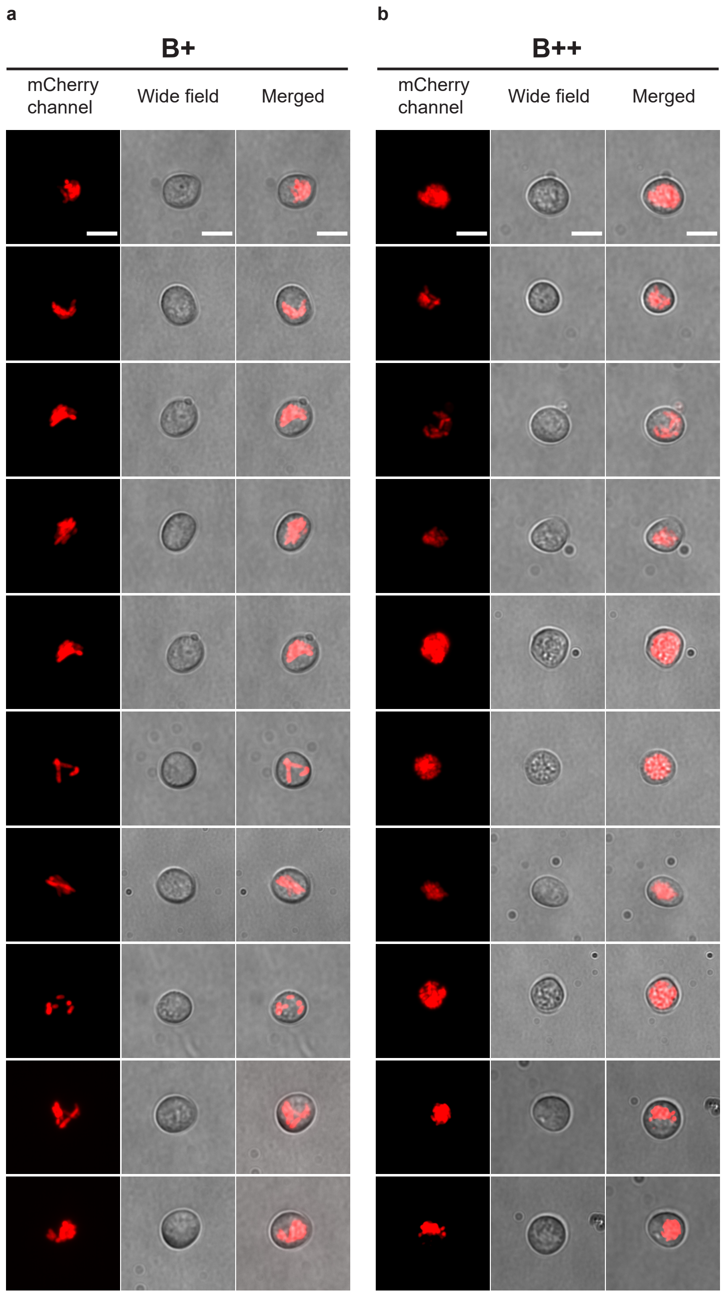

Supplementary Figure 4: Subpopulations of B+ and B++

a -b, Images of FACS-sorted B+ (a) and B++ (b) spores with intracellular bacteria after vertical transmission. The images show an overlay of two-dimensional projections of the wide-field and mCherry-signal z-stack. Scale bars, 5 µm. Source data are provided as a Source Data file and imaging data on zenodo (ref[69]).

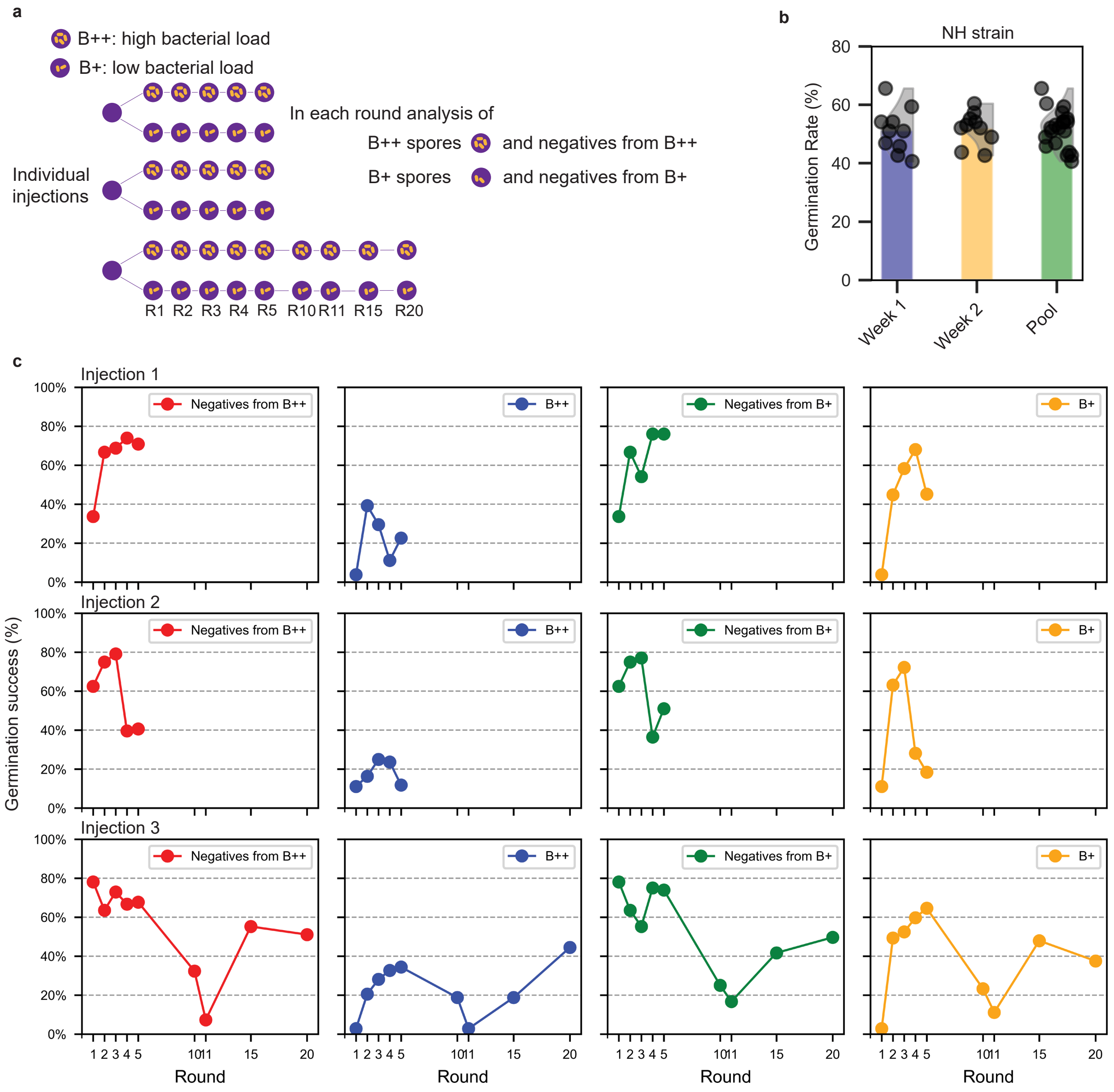

Supplementary Figure 5: Development of germination success across individual injections with germination baseline of the NH strain

a, Scheme of Selection regime: In each round the arising spores will have both positive (B++ and B+) and negative (B-) spores, germination success is determined for both population each. **b**, the baseline of the germination success for the non-host strains was determined equally to the passaging regime for two weeks (n = 10). **c**, Germination success data from three individual injections. Source data are provided as a Source Data file and FACS data on zenodo (ref[69]).

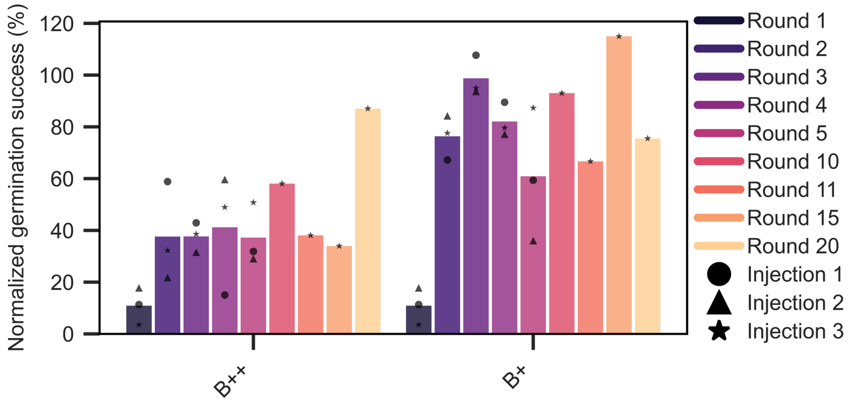

Supplementary Figure 6: Normalized germination success across three injections

In each round the germination success of bacteria positive germlings was normalized by the germination success of bacteria negative germlings originating from the same source plate; Raw data for each line is depicted in Figure 2 and Supplementary Figure 4. Source data are provided as a Source Data file.

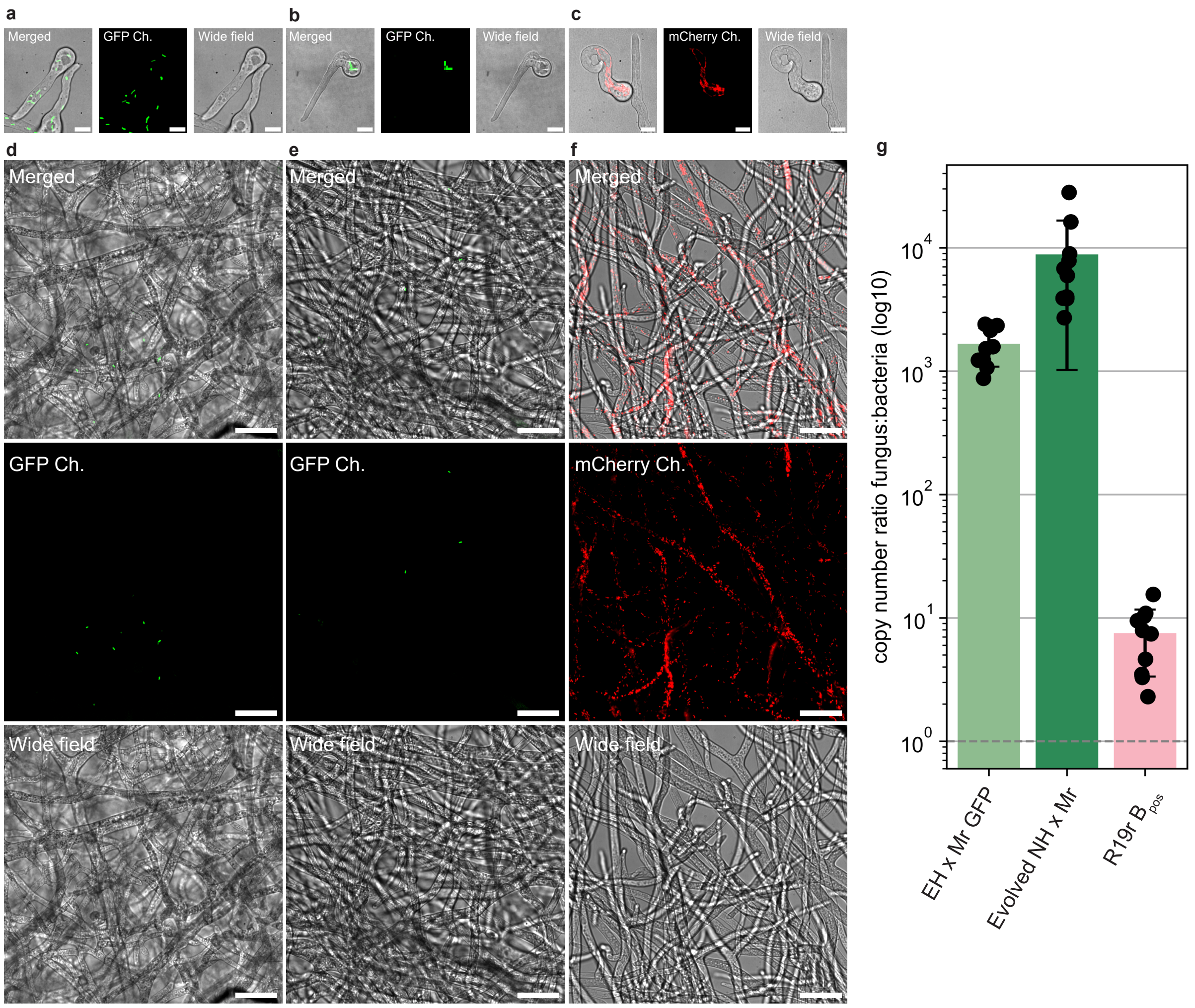

Supplementary Figure 7: bacterial load detection of EH and NH strains with *Mycetohabitans rhizoxinica* and *Ralstonia pickettii*

The colonization of the fungus by *Ralstonia pickettii* exceeds the one by *Mycetohabitans rhizoxinica*. **a-b**, pictures of young germlings of cultivated in liquid potato dextrose broth (PDB) medium showing that colonization of the EH strain (**a**) and the NH strain (**b**) by the natural endosymbiont *M. rhizoxinica* is apparently lower than by *R. pickettii* after germination (**c**); **e-f**, images of mature mycelium originating from single positive spores of EH colonized by its natural endosymbiont *M. rhizoxinica* (**d**), NH with *M. rhizoxinica* (**e**) and NH with *R. pickettii*. (**g**), bacterial colonization obtained by qPCR shows that *R. pickettii* (recovered round 19 B_{pos} spores) reaches larger colonization levels than *M. rhizoxinica* in the EH and NH strain; shown are averages of log10 transformed copy number ratios of fungal to bacterial copies from 9 (Evolved NH x Mr) to 10 (EH x Mr GFP and R19r B_{pos}) biological replicates ± standard deviation, low values indicate high bacterial colonization. Source data are provided as a Source Data file and imaging data on zenodo (ref[69]).

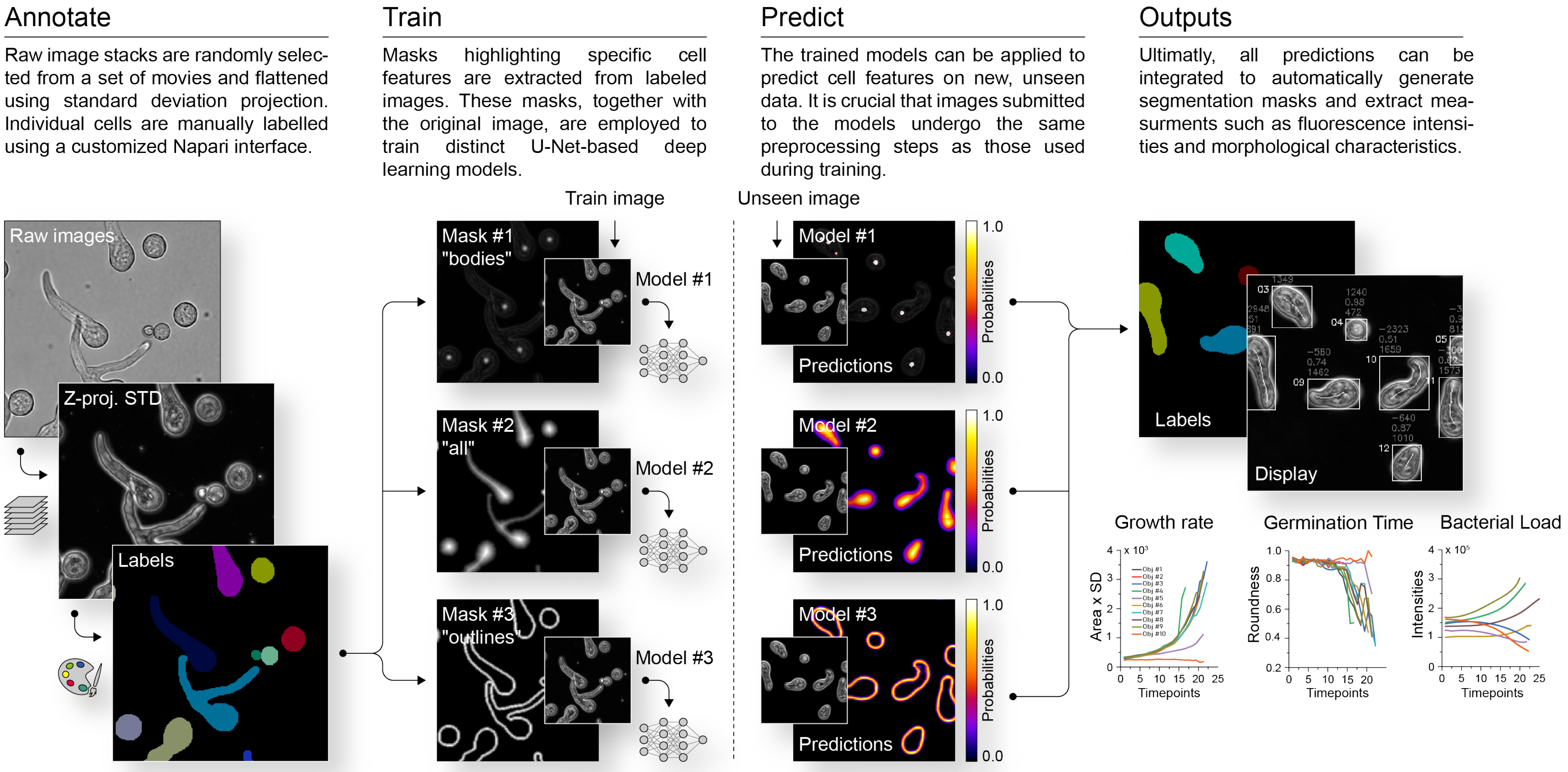

Supplementary Figure 8: Graphical workflow of deep-learning model for fungal image analysis.

The tool developed here allows to automatically analyze germination timelapse videos to track fungal growth, germination time and internal bacterial load. The code for this tool and annotated training images are available on GitHub (https://github.com/BDehapiot/ETH-ScopeM_Gassler).

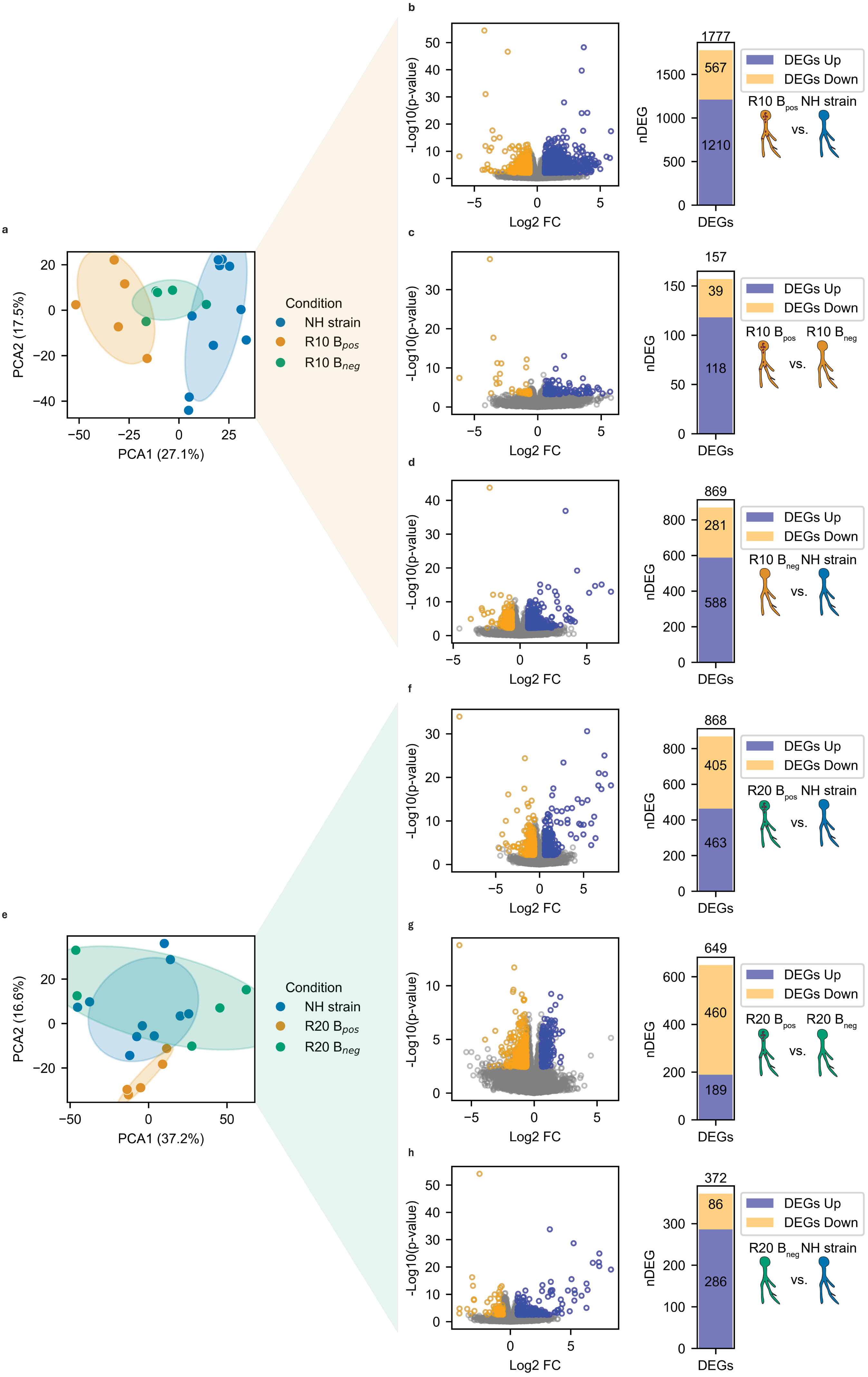

a
PCA2 (17.5%)
PCA1 (27.1%)
Condition
NH strain
R10 B$_{pos}$
R10 B$_{neg}$
b
-Log10(p-value)
Log2 FC
nDEG
DEGs
1777
567
1210
DEGs Up
DEGs Down
R10 B$_{pos}$ NH strain
vs.
c
-Log10(p-value)
Log2 FC
nDEG
DEGs
157
39
118
DEGs Up
DEGs Down
R10 B$_{pos}$ R10 B$_{neg}$
vs.
d
-Log10(p-value)
Log2 FC
nDEG
DEGs
869
281
588
DEGs Up
DEGs Down
R10 B$_{neg}$ NH strain
vs.
e
PCA2 (16.6%)
PCA1 (37.2%)
Condition
NH strain
R20 B$_{pos}$
R20 B$_{neg}$
f
-Log10(p-value)
Log2 FC
nDEG
DEGs
868
405
463
DEGs Up
DEGs Down
R20 B$_{pos}$ NH strain
vs.
g
-Log10(p-value)
Log2 FC
nDEG
DEGs
649
460
189
DEGs Up
DEGs Down
R20 B$_{pos}$ R20 B$_{neg}$
vs.
h
-Log10(p-value)
Log2 FC
nDEG
DEGs
372
86
286
DEGs Up
DEGs Down
R20 B$_{neg}$ NH strain
vs.

Supplementary Figure 9: Principal component analysis of individual differential gene expression comparisons

a and **e**, Principal component analysis (PCA) of gene count data from RNA sequencing shows that global expression profile in early round 10 (a) and late stage round 20 of adaptation (b) with NH-strain, bacteria positive (B_{pos}) and bacteria negative (B_{neg}) samples, samples originated from the low bacterial load adaptation line. **b** to **d** and **f** to **h**, Differenttial gene expression analysis between different comparions including number of differnitaly expressed genes displaying significantly up- (blue) and downregulated (yellow) genes (significance cut-off values: Log2 Fold Change > 0.6 (~1.5 abosulte fold change) and p-value < 0.05 (adjusted according to Hochberg in DeSeq2), grey marked genes are below the significance threshold. Source data are provided as a Source Data file.

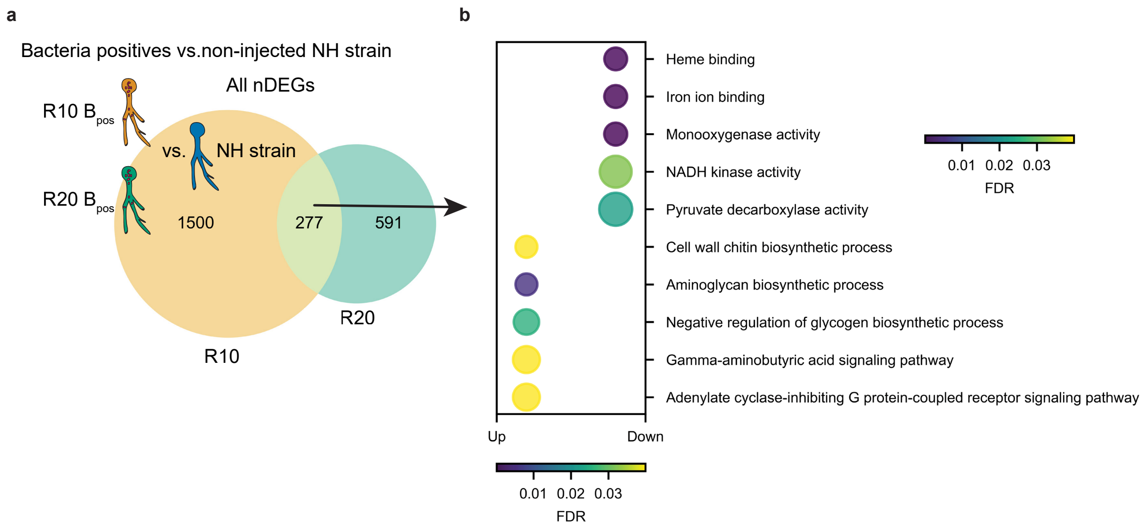

Supplementary Figure 10: Gene ontology (GO) term enrichment of bacteria positive samples versus non-injected (NH-strains)

a, Overlap of differentially expressed genes between R20 B_{pos} vs. NH-strain and R10 B_{pos} vs. NH-strain; **b**, Enrichment analysis by biological process on genes which are significantly upregulated in both data sets (277 genes). String anaylsis with up- and downregulated genes was performed against the upladed NH proteome (STRG0A18OCV), bubble sizes indicate strength of each term (i.e. Log10 of observed to expected ratio of gene counts, value multiplied by a constant factor for visualization) and buble colors indicate the false discovery rate. Source data are provided as a Source Data file.

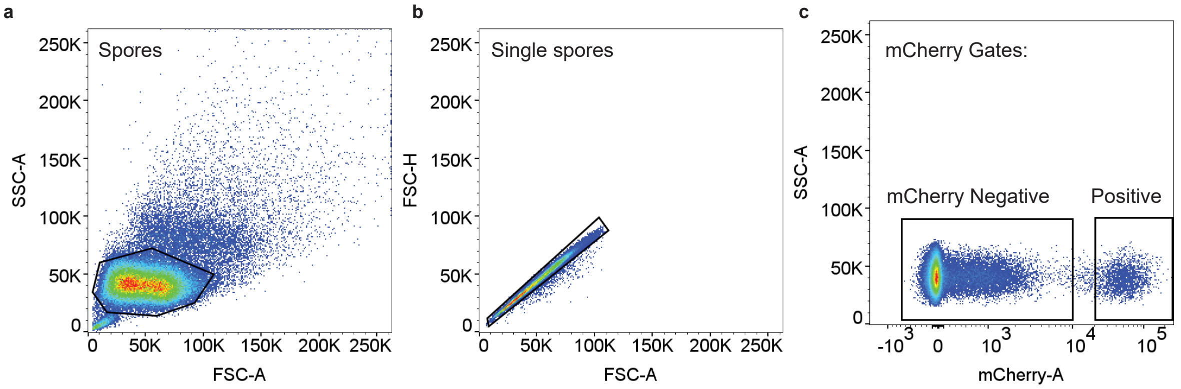

Supplementary Figure 11: Gating Strategy for FACS analysis

Depiction of general gating strategy for flow cytometry analysis and cell sorting. **a-b**, Single spores were selected through side scatter (SSC) versus forward scatter (FSC) gating using (**a**) SSC-A vs. FSC-A followed by (**b**) FSC-H vs. FSC-A gates. **c**, The presence of bacteria was checked by SSC vs. fluorescence gating using an SSC-A vs. mCherry-A gate. All FACS data are uploaded on zenodo (ref[69]).
