# Supplementary material for: Induced endosymbiosis between a fungus and bacterium reveals a shift from antagonism to commensalism
Source: Nat Commun. 2025 Nov 28;16:10717. doi: 10.1038/s41467-025-65741-9 (PMC12663227; doi:10.1038/s41467-025-65741-9)
Supplement: Supplementary file 2 — Description of Additional Supplementary Files [file 41467_2025_65741_MOESM2_ESM.pdf]

Description of Additional Supplementary Files:

Supplementary Data 1: Top ten up- and downregulated genes and StringDB permalinks for all reported subsets

Supplementary Data 2: This table lists genes from Lastovetsky et al. (https://doi.org/10.1128/mbio.02088-20) that are also present in the differentially expressed genes stemming from the comparison between Bpos R20 vs. Bpos R10.

Supplementary Data 3: List of strains and chemicals used in this study.

Supplementary Video 1: Cytosolic growth of *R. pickettii* within *R. microsporus* NH strain after injection. The video is an overlay of the brightfield image in grey and the contrast-adjusted 2D projection of the z-stack of the mCherry signal in red, scale bars = 20 µm. The video shows a single injected bacterium that grows in the cytosol of the recovering germling.

Supplementary Video 2: Movement of *R. pickettii* within *R. microsporus* NH strain after injection. The video is an overlay of the brightfield image in grey and the contrast-adjusted 2D projection of the z-stack of the mCherry signal in red, scale bar = 20 µm. The bulk flow in the mycelium can be observed through the motion of large cytosolic compounds. The bacteria can be observed to move only in the direction of pressure-induced bulk flow.

Supplementary Video 3: Germination of *R. microsporus* NH strain after injection with *R. pickettii*. The video is an overlay of the contrast-adjusted z-stack 2D projection of the brightfield image in grey and the mCherry signal in red, scale bar = 20 µm.

Supplementary Video 4: Germination of *R. microsporus* NH strain spores after injection with *R. pickettii* and serial passaging over 20 rounds under the low bacterial load regime. The video is an overlay of the contrast-adjusted z-stack 2D projection of the brightfield image in grey and the mCherry signal in yellow, scale bars = 10 µm.
